# Food Sharing among Hadza Hunter-Gatherer Children

**DOI:** 10.1371/journal.pone.0131996

**Published:** 2015-07-07

**Authors:** Alyssa N. Crittenden, David A. Zes

**Affiliations:** 1 Department of Anthropology, University of Nevada, Las Vegas, Las Vegas, Nevada, United States of America; 2 Department of Ecological Statistics, University of California Los Angeles, Los Angeles, California, United States of America; Universidad Carlos III de Madrid, SPAIN

## Abstract

Human prosociality is one of the defining characteristics of our species, yet the ontogeny of altruistic behavior remains poorly understood. The evolution of widespread food sharing in humans helped shape cooperation, family formation, life history, language, and the development of economies of scale. While the behavioral and ecological correlates of food sharing among adults are widely studied, very little is known about food sharing among children. Here, in the first study to analyze the food sharing patterns of hunter-gatherer children, we show that while sharing may be biased towards kin, reciprocity characterizes the majority of all sharing dyads, both related and unrelated. These data lend support to the recent claim that discrimination among kin might be linked with reciprocal altruism theory. Furthermore, we show that age positively correlates with an increase in sharing, both in frequency and amount, supporting recent suggestions that prosocial behaviors and egalitarianism develop strongly in middle childhood when children acquire the normative rules of their society.

## Introduction

The evolution of routine food sharing outside of the mother-infant dyad is one of the fundamental characteristics that differentiates humans from other apes [1]. Sharing food may function to reduce variance in consumption, develop and maintain social bonds, or advertise skill in resource acquisition [2–7]. The importance of food sharing is implicit in evolutionary models of life history, family formation, and altruism [8–12], yet the ontogeny of such behaviors is conspicuously absent from most theoretical models (for a notable exception, see [13]). Our long, slow, and dynamic childhood is a defining characteristic of our species’ life history. Given that childhood is argued to be the time when other-regarding preferences and egalitarianism develop [14], it is crucial to begin incorporating children into our evolutionary models in order to understand how the development of food sharing may influence prosocial behaviors.

Foraging populations, who share food on a daily basis, are ideal for studying the social, ecological, and nutritional components of food distribution and consumption [3,5,11]. A wide range of evolutionary models explaining food sharing among hunter-gatherers has been proposed. Two of the most prominent models are “kin selection” and “reciprocity”. The kin selection model proposes that an individual should be willing to share as long as the cost of sharing is less than the benefit, times the coefficient of relatedness between individuals C < B x r [15]. Kin selection is a process where genes are favored because of their potential beneficial effects on the fitness of close relatives who share many of the same genes by descent (e.g. inclusive fitness). Behavioral data has demonstrated that individuals in small-scale societies may preferentially assist close kin over more distantly related kin and non-kin [16,17]. Recently, however, mounting data suggests that altruism leading to cooperation might not be the product of shared genes alone and that individuals might give preferential treatment to relatives over nonrelatives if those individuals possess traits that are preferred in food sharing or social partners [18]. These data are associated with the reciprocal altruism model that is based on the premise that givers and receivers reverse positions on a systematic basis and amounts given and received are correlated over time where the benefit to the recipient is greater than the cost to the giver [19–21]. Here, we determine whether Hadza hunter-gatherer children are selecting sharing partners based on kinship or reciprocity, and determine if the frequency of sharing and/or amount shared (kilocalories) differs between related and unrelated partners.

The Hadza are a group of mobile foragers living in Northern Tanzania, south of the Serengeti, in a savanna woodland environment [22]. Approximately 250 individuals, out of a total population of 1000, practice hunting and gathering as their almost exclusive means of subsistence. They live in camps with roughly 30 individuals, however camp composition is fluid and frequent movement between camps is common [23]. Camps move approximately every two months in response to the seasonal availability of resources [24], as the Hadza diet consists of a wide variety of plant and animal foods. Men typically forage alone or in pairs, focusing their foraging efforts primarily on honey collection and hunting a wide variety of birds and mammals. Women forage in groups and primarily focus on gathering plant foods such as baobab fruit, berries, and tubers (underground storage organs). Nursing infants accompany their mothers on daily foraging trips until they are approximately 2–3 years old and being weaned. Once children are weaned (on ground baobab powder, animal fat, pre-masticated meat, and/or liquid honey), they are typically left in camp without adult supervision but may remain under the charge of at least one elderly camp member or an older juvenile caregiver [25]. Once they reach around three years of age, they spend ample time foraging [25]; they may accompany their mothers on short foraging excursions or forage close to camp in mixed age and sex parties of children. Girls almost always forage in groups, as do adult women, whereas boys begin to forage alone or in pairs, like adult men, around the age of ten years. Hadza children are particularly dynamic foragers, even by hunter-gatherer standards. The earliest ethnographic reports of Hadza children reported high levels of productivity [26]. In the 1980s and early 1990s, a team led by Blurton Jones, Hawkes, and O'Connell quantified Hadza child economic contributions and estimated return rates for a subset of foraged foods. Their data suggested that Hadza children are very active foragers and can collect up to 50% of their daily energy requirement above the age of five years [27]. More recently, a meta-analysis by Crittenden and colleagues documented the full complement of Hadza children's foods and provided detailed foraging, food return, and consumption data for a large sample of Hadza children living in different regions of Hadza territory [28]. These results confirmed child productivity and showed that children focus their collection efforts on the following categories of foods in descending order: fruit (e.g. baobab, berries, figs), birds, tubers, honey, small game (e.g. hyrax, bush mice, galagos), and drupes and legumes [28]. In addition to being avid foragers, Hadza children routinely share their food with friends and family.

Hadza children grow up in an egalitarian community where food sharing is central to group formation and survival. They may enter into food sharing relationships with hundreds of individuals throughout their lifetimes [29] and sharing food may act to build and maintain social bonds. In an attempt to learn more about children’s food sharing decisions, we analyzed mid-day meals—which typically occur without adult supervision and in communal spaces away from home hearths where children assemble and play. During these typical mid-day meals, children eat while they play and are free to distribute their collected food as they choose with no adult oversight over distribution. For this reason, we were able to analyze partner choice to determine whether kin selection or reciprocity appears to characterize the selection of food sharing partners among Hadza children.

## Materials and Methods

### Ethics Statement

Human Research Subjects approval was obtained from the University of California, San Diego Human Research Subjects Institutional Review Board and the Committee on the Use of Human Subjects at Harvard University. Oral informed consent was obtained from all participating children and their parents. Participant consent was recorded and witnessed affidavits were obtained. As the Hadza are not a literate population, oral consent was necessary. The IRB offices at both universities and the necessary Tanzanian government agencies approved the consent procedure. All data were collected with the permission of the Tanzanian Commission for Science and Technology (COSTECH).

### Data Collection

Data were collected in two Hadza bush camps over a combined total of 36 days during 2005 and 2006. The study was conducted in camps located in the area (approximately 20,000 hectares) recently deeded to the Hadza in 2011 by the Tanzanian government for traditional uses, including hunting and gathering [30]. Currently, although the land is legally titled to the Hadza, it remains communal and continues to be utilized by pastoral tribes in the area.

We collected three types of data: (1) ad libitum naturalistic sharing data on 62 meals containing 128 instances of dyadic food sharing, (2) ethnographic interviews of children and adults to determine whether or not children maintain producer control over their foraging yield, and (3) instantaneous scan observations from sunrise to sunset (13 per day every hour on the hour from 7am to 7pm) in which the behavior of every individual in camp was recorded. By analyzing these in-camp data, we were able to control for presence at each meal, regardless of whether or not they participated in a sharing event, therefore excluding the possibility that sharing is biased by differential presence at meals. All children were present in camp during every meal but two and were therefore available to share or be shared with—and yet only a subset of these actors participated in food transfers. The recorded meals took place without any adult supervision in communal spaces in the center of the camp where the children convened and played during the day. We conducted semi-structured interviews with both children and adults to determine if children had producer control over the food that they returned to camp with. Adults and children alike responded that during mid-day meals, children were free to distribute their foraging yield as they pleased. As adults are rarely in camp when children return from foraging, they do not control the distribution of children’s food during the mid-day meals. The role that parents and other individuals play in the process of cultural transmission, however, cannot be discounted [31, 32].

A total of 36 children were in residence, however our sample includes only 35 individuals (age 2–17 years; 13 males and 22 females); we removed individuals who appeared in less than ten scans, which allowed us to exclude visitors or children who were frequently out of camp during mid-day meals and thus unavailable for participation in a sharing event, defined as either giving or receiving food. Of the total 106 sharing dyads present in the sharing network, 54 were kin members and 52 were unrelated. We estimated kilogram wet weight of each food based on measured reference samples for each food type and then converted into caloric values. The energy values for all foods were determined using standard analytical methods for wild foods [33,34].

Kin relatedness was determined by interviews conducted in each camp. Ages were visually estimated and then corroborated with long-term census data from Nicholas Blurton Jones (1982–1994), Frank Marlowe (1995–2010), and Alyssa Crittenden (2006 –present).

### Statistical Inference

Due to the unknown correlation structure among dyads and because of possible non-Gaussian (non-linear) responses, we used two non-parametric tests of statistical significance: QAP (quadratic assignment procedure), detailed below, and permutation testing which provided simulations of the null distributions of the test statistic of interest. We did not perform a model-based analysis because non-parametric analyses, although comparatively conservative, require far less assumptions about meal size, partner selection, or frequency of sharing. Using QAP allowed us to use naturalistic data free from such restrictive assumptions.

### Quantification

Percent of time present at meals was calculated by dividing the number of observations present at a meal by the total number of scans in which an individual appeared. While only a subset of the individuals participated in food transfers, using a goodness of fit test, we found no evidence of significant differential presence of individuals at observed meals (χ2 Camp 1, p = .91; Camp 2, p = 0.58). The aim of our project was to determine who was sharing and what might be motivating their resource distribution choices, therefore, we neither collapsed across all children, nor did we calculate average number of transfers per child. If we were to average across all children in the sample, we would collapse all of the variation and subsequently be unable to determine whether children share more with related or unrelated individuals.

When analyzing food transfers, the raw response of interest was quantity (i.e. kilocalories) of a meal shared. Kilocalories, a heavy right-skewed variable, were log-transformed to help robustize inference, and also, importantly, to avoid a few large observations from biasing results. For example, the sum of log kilocalories shared for an individual, *i*, was calculated as Σ*j log* [y(*i*, *j*) + 1], where *y(i*,*j)* was the meal size in kilocalories given by *i* to individual *j*, with *j* indexing across all other individuals.

We quantified kinship using degree of relatedness (DOR), 0.5 for siblings; 0.25 for half-siblings, aunts, and uncles; and 0.125 for cousins. For each camp we constructed a square symmetric adjacency matrix, *X*, containing DOR values. A corresponding response adjacency matrix, *Y*, was constructed such that the scalar value at row *i*, column *j*, contained the sum log kilocalories or frequency of a meal given by child *i* to child *j*. The two predictor matrices, *X*_*1*_, *X*_*2*_, (one for each camp) were vectorized and united to create a single vector, *x*, of DOR values. Likewise, we constructed *y* from *Y*_*1*_ and *Y*_*2*_. The observed correlation between DOR and sharing was then = *s_xy_/s_x_s_y_*. The null distribution of the random variable *r* was estimated using QAP. The columns of the two response adjacency matrices were randomly shuffled to create two null responses, π(*Y*_*1*_) and π(*Y*_*2*_), that were then vectorized to create π(*y*). The correlation between *x* and π(*y*) is an example of the random variable *r* under the null hypothesis. By iterating this process (50,000 times), the *p*-value was determined as the ratio of the total number of null *r*’s that are equal to or exceed the observed *r* over the total number of iterations.

### Reciprocity

We measured reciprocity as the correlation between total amount shared from child *i* to child *j* and total amount shared from child *j* to child *i*. As with kinship, *Y*_*1*_ and *Y*_*2*_ were the response adjacency matrices of total log kilocalories shared or frequency of shares. Where *u* was the vector made from the upper triangles of *Y*_*1*_ and *Y*_*2*_, and *w* was the vector of the corresponding lower triangles. The observed reciprocity was = *s*_*uw*_/*s*_*u*_ *s*_*w*_. We estimated the random variable *r* under the null through the correlation between *u* and π(*w*), where π(*w*) is the vector of lower triangle entries of π(*Y*_*1*_) and π(*Y*_*2*_).

### Age Effects

We constructed a data frame with observational units consisting of all children that participated in a sharing dyad (i.e. giving or receiving food). A variable was created quantifying the total log kilocalories shared by each child. This response was then correlated with age or, in the case of differential effects, the difference between the age of the giver and the age of the receiver. For inference, the null sampling distribution was simulated through permutation testing.

## Results and Discussion

### Kin Selection vs. Reciprocity

We find that children who are genetically related tend to share food in higher frequency (frequency pooled, correlation by QAP, one-sided: 0.2289222 –p = 2e-04) and in greater amounts (log kcals pooled, by QAP, one-sided: 246.7923 –p ≈ 0) when compared to sharing between unrelated children. In addition, a higher degree of genetic relatedness is correlated with greater amount shared (r = 0.1420186, p = 0.00016, one-sided QAP on correlation) and a higher frequency of sharing (r = 0.1629569, p ≈ 0, one-sided QAP on correlation). When analyzing overall amount shared, we find that reciprocity characterized the majority of *all dyads* (log kcals pooled, correlation by QAP: 0.2354221 –p = 0.0014), including related and unrelated sharing partners.

### Age and Sex Effects

**Fig 1:**
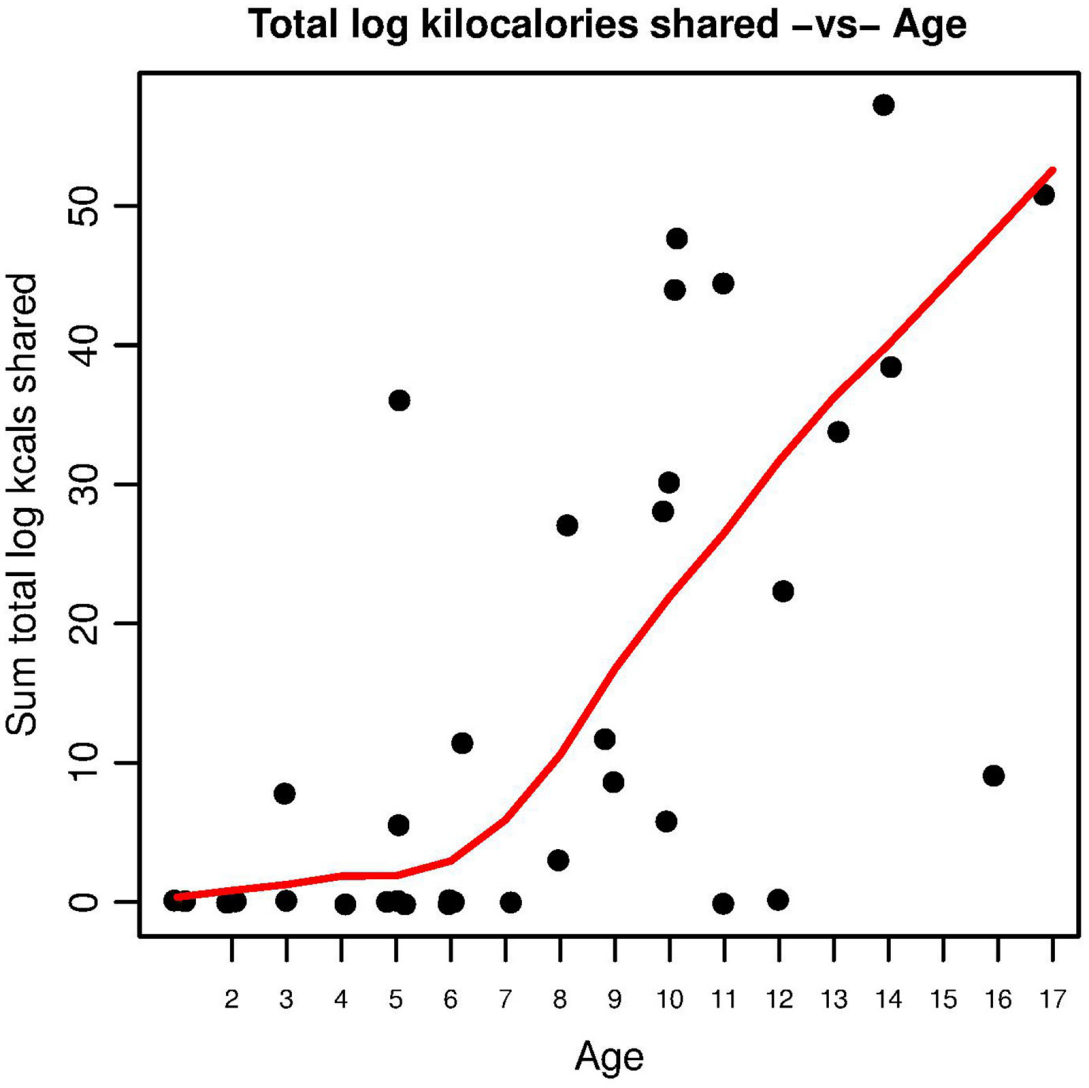
Food sharing (measured as kilocalories given) as a function of age.

Among the Hadza, the frequency of food transfers increases with age commencing at approximately age 7–8 (Fig 1). There is a correlation between increase in age, total amount of food shared (*r* = 0.637, *p* < 0.001, one-sided permutation test on correlation), and frequency of giving (*r* = 0.608, *p* < 0.001, one-sided permutation test on correlation). We find no correlation between amount given and the age difference between giver and receiver (*r* = -0.104, p = 0.888, one-sided permutation test on correlation), suggesting that older children are not preferentially sharing with younger children.

Although age appears to be a strong predictor of amount shared, we find no such evidence of sex differences in amount of food shared (*r* = 0.0014, *p* = 0.503, two-sided permutation test on correlation).

## Conclusion

The data presented here suggest that children begin sharing food at very young ages. The development of food sharing networks in childhood suggests that altruistic behavior in humans develops early. Our results indicate that Hadza children routinely share food—from snack size to meal size—with both related and unrelated children in camp and sharing increases with age. While sharing appears to be biased towards kin, a causal relationship between relatedness and sharing is not necessarily confirmed, as food sharing by kin may derive from the fact that they co-reside. Future work will aim to tease apart these effects.

Reciprocity characterizes all sharing dyads, suggesting that the substantial food sharing that occurs between children is, at least in part, motivated by reciprocity. These data support recent claims that discrimination among kin might be linked with reciprocal altruism theory [18], and that reciprocity appears to be a common predictor of food sharing behavior [35]. The strong correlation between increase in age and increase in amount of food shared supports the hypothesis that other-regarding preferences and egalitarianism develop in middle childhood [36, 37], coinciding with the time when children begin to incorporate the normative rules of the society in which they live [14]. The development of food sharing relationships in childhood can further function to counteract or lessen the free-rider problem, which may be limited by small population size [38]. This appears to be true among Hadza children where population size is small and free riders, therefore, have longer search time in order to exploit new individuals.

Sharing is likely involved in the social construction of relationships at a very early age and may function to establish friendships and cement social bonds. Over their lifetimes, Hadza children interact with the same core group of individuals and social bonds are reinforced with the sharing of food. In addition to strengthening friendships, any assistance provided to children, even in modest caloric amounts, helps to subsidize their growth by alleviating temporary fitness costs. Future work will explore the mechanisms by which Hadza children learn to share, including processes of social learning and modes of transmission. In order to more fully grasp how food sharing influenced the evolution of human life history, family formation, and the development of prosocial behaviors, we must begin to take into account the complexities of the social networks of children and explore the ontogeny of food sharing.
